# Emotional and behaviour difficulties in teenagers with permanent childhood hearing loss

**DOI:** 10.1016/j.ijporl.2017.07.031

**Published:** 2017-10

**Authors:** Jim Stevenson, Hannah Pimperton, Jana Kreppner, Sarah Worsfold, Emmanouela Terlektsi, Colin Kennedy

**Affiliations:** a Faculty of Social and Human Sciences, University of Southampton, Southampton, UK; b Faculty of Medicine, University of Southampton, Southampton, UK; c School of Education, University of Birmingham, UK

**Keywords:** Permanent childhood hearing loss, Emotional and behavioural difficulties, Receptive language, Longitudinal study, Teenagers

## Abstract

**Objectives:**

It is known that during the middle childhood years those with permanent childhood hearing loss (PCHL) are at increased risk of showing emotional and behaviour difficulties (EBD). It has yet to be established whether this risk continues into the late teenage years. There is a paucity of longitudinal studies on the association between PCHL and EBD.

**Methods:**

The Strengths and Difficulties Questionnaire (SDQ) was used to measure EBD based on parent, teacher and self-ratings in 76 teenagers with PCHL and 38 in a hearing comparison group (HCG) from a population sample of children that was followed up from birth to adolescence.

**Results:**

On parent-rated SDQ, the PCHL group had significantly higher Total Difficulties score than the HCG (Standardised mean difference (SMD) = +0.39, 95%CI 0.00 to 0.79). Amongst the PCHL group the presence of disabilities other than hearing loss had a substantial impact on the level of parent-rated EBD (SMD = +1.68, 1.04 to 2.33). There was a relationship between receptive language ability and EBD in both the HCG (r = −0.33, 95%CI −0.59 to −0.01) and the PCHI group (r = −0.33, 95%CI −0.53 to −0.02). The effect of PCHL on EBD became non-significant when receptive language was included as a covariate (F = 0.12, df = 1,95, p = 0.729). Early confirmation of hearing loss (i.e. before 9 months of age) did not have a significant effect on EBD scores (SMD = +0.31, 95%CI −0.15 to 0.77).

**Conclusions:**

PCHL continues to be associated with elevated EBD scores as measured by parent rated SDQ into the late teenage years but the degree of this elevation is less than in childhood and is not apparent on teacher or self-ratings. Poor receptive language ability appeared to account for these elevated EBD scores in the group with PCHL. Particular attention needs to be paid to the mental health of children and adolescents with PCHL that is accompanied by other disabilities and to those with poor receptive language ability. However, the majority of teenagers with PCHL do not show clinically significant elevated levels of EBD.

## 1 Introduction

There is well-replicated evidence that in childhood those with permanent childhood hearing loss (PCHL) are at an increased risk of poor psychosocial functioning [1], [2]. These difficulties are apparent from the preschool age range [3]. Adolescents with PCHL face a number of challenges not experienced by their hearing peers and can find some aspects, such a friendship and peer relations, particularly daunting [4]. Children with PCHL are likely to be at risk of developing emotional and behaviour difficulties (EBD) as a result of a number of factors. Their social-emotional development may be adversely influenced by difficulties in communication and many have additional cognitive and physical impairments that are risk factors for EBD [5], [6], [7]. For this reason it is important to determine if, and to what extent, they show an elevated rate of mental health problems compared to children in the general population so that intervention can be targeted at this potentially vulnerable group.

A narrative review linked hearing loss (HL) with mental health problems in children and adolescents, including depression, aggression, oppositional defiant disorder and conduct disorder, and, less consistently, anxiety, somatization, and delinquency [1]. That review was based on 35 papers and found that there were substantial differences between studies and marked heterogeneity in the HL population. The review commented on the absence of longitudinal studies on this issue.

A quantitative review of studies on the mental health of children with HL presented results in two parts [2]. The first part identified 33 studies in which emotional and behaviour difficulties in children with HL could be compared to a hearing comparison group (HCG) using a variety of measures of EBD. The average effect size (standardised mean difference, SMD) for these studies was 0.36. The second part reported a meta-analysis on 12 studies of children with HL using the Strengths and Difficulties Questionnaire (SDQ) [8]. The estimated effect sizes were +0.23 (95%CI 0.07 to 0.40), +0.34 (95%CI 0.19 to 0.49) and −0.01 (95%CI −0.32 to 0.13) for parent, teacher and self-report ratings of Total Difficulties respectively. The most consistent differences between children with HL and a HCG were in the area of Peer Problems.

In that meta-analysis based on cross-sectional studies of children with HL at different ages, there was no evidence of age-related changes in the risk of EBD in children with HL. There is a paucity of evidence regarding age-related changes from longitudinally studied samples. One such study of children with “hearing problems”, identified on the basis of parental report either at age less than 1 year or at age 4–5 years [9], assessed children on the SDQ for EBD at ages up to 10–11 years. There was no clear pattern of an increasing or decreasing level of EBD over time. There is a need to extend such longitudinal studies into the adolescent years and on the basis of HL established using objective audiological evaluation.

There are aspects of development and management that may be related to EBD score in those with PCHL. When the PCHL sample in the present study was assessed at age 6–10 years, there was a strong relationship between EBD scores and poor expressive and receptive language ability [10]. However we found, at that age, that while early confirmation of PCHL had a beneficial effect on receptive language development, it had no significant impact in reducing behaviour problems in children with PCHL [11]. We argued that this beneficial effect of early confirmation on language development at age 6–10 years was not sufficiently great to bring the language ability of this early confirmed group to the level of the hearing comparison group (HCG) and therefore the increased risk of behaviour problems remained. Early confirmation also had a beneficial effect at ages 13–20 years on reading comprehension [12], though significant benefits of early confirmation on receptive language ability were only detectable for those without cochlear implants [13]. There was no significant benefit for language development [13] or for reading [12] from exposure to Universal Neonatal Hearing Screening (UNHS). It is possible that by this later age any enhancement of language development by early confirmation may be sufficient to have an impact on behaviour.

Here we present the findings from a longitudinal study whose participants were assessed in their teenage years. We previously reported a number of findings in infancy and the first decade of life in this sample [14], [15], [16]. The first aim of the present study was to examine whether the pattern of elevated SDQ scores, indicating the presence of behaviour problems, demonstrated in the group with PCHL in childhood was still present in the teenage years. The second aim was to determine which factors, including early confirmation of PCHL, exposure to UNHS, cochlear implantation (CI), poorer receptive language ability, and/or the presence of other disabilities, were associated with high EBD scores within the PCHL group.

## 2 Methods

### 2.1 Participants

The study was a population-based cohort study of children with bilateral PCHL that also included a HCG that was half the size of the group of participants with PCHL. The 183 adolescents aged 13–20 years (120 with PCHL, 63 in the HCG) who were eligible for this prospective follow-up study were drawn from a birth cohort of children born in eight districts of southern England and had participated in a previous phase of the study aged 6–10 years [14], [15], [16]. The birth cohort comprised two sub-cohorts: First, the 1993–1996 Wessex birth cohort of 54,000 babies enrolled in the Wessex controlled trial of UNHS [17], Second, the 1992–1997 Greater London birth cohort of 100,000 babies, born in four districts in Greater London, of which two were the only two districts in the UK (Waltham Forest; Hillingdon) that provided UNHS for PCHL in the early 1990s and the other two were districts geographically adjacent to them (Redbridge; Brent & Harrow, respectively). The adolescents with PCHL had all been diagnosed with PCHL ≥40 dB in the better ear that was not known to be acquired. The HCG was comparable to those with PCHL with respect to place and date of birth.

At the earlier time point in childhood (Time 1), 120 participants with PCHL and 63 in the HCG took part at a mean (SD) age of 7.96 (1.23) years. At the second time point (Time 2) 76 teenagers with PCHL and 38 in the HCG participated in the current study at a mean age of 16.84 (1.42) years. The annual attrition rate among children with PCHL eligible for the present study was 3% over 17 years since their exposure (or not) to UNHS and 4% over the 9 years since their assessment at primary school. Attrition was largely attributable to the participants not responding to requests to participate in later phases of the study.

Severity of hearing impairment was categorised from the most recent audiological evaluation at audiology and cochlear implant clinics as moderate (40–69 dB HL), severe (70–94 dB HL) or profound (≥95 dB HL) according to four-frequency averaging of the pure-tone thresholds at 0.5, 1, 2 and 4 kHz in the better ear. It should be noted that the PCHL group is an unselected population of all children with bilateral PCHL of ≥40 dB and that those with severe and profound PCHL make up only half of the sample with the remainder having been diagnosed with moderate PCHL.

Other disabilities in addition to PCHL included cerebral palsy, visual impairment, and learning disability. The latter was determined by a Ravens Progressive Matrices score equivalent to a non-verbal IQ less than 70 [18]. The presence of conditions was noted from medical records and parent report.

This study was approved by the Southampton and SW Hampshire Research Ethics Committee. Written informed consent for participation in the study was obtained from principal caregivers and from the teenage participants themselves.

### 2.2 Measures of EBD

EBD were measured with teacher, parent and self-report versions of the Strengths and Difficulties Questionnaire (SDQ) [8]. This is a widely used behaviour screening questionnaire on children and young people's behaviours, emotions, and relationships. It has been recommended as suitable for use with children with PCHL [19], [20]. Total Difficulties score reflecting EBD in the child was derived from summing the scores of four SDQ scales (Emotional Symptoms, Conduct Problems, Hyperactivity, and Peer Problems) in the parent, teacher and self-report questionnaires separately. Higher Total Difficulties scores indicate more EBD. A fifth scale measured Prosocial Behaviour on which higher scores indicate more prosocial behaviour.

The self-report SDQ has been found to be a less good predictor of psychiatric diagnosis than parent and teacher ratings [21] and there have been reports that the psychometric characteristics of the self-rated SDQ are less than optimal in relation to scale reliabilities [22] and to item loadings [23]. Teacher rated SDQ scores were obtained on less than 75% of the participants. For these reasons the report of the findings on the teacher-rated and self-rated SDQ scores will be limited to an initial comparison between the HCG and PCHL groups at Time 2.

### 2.3 Measures of non-verbal ability and language

For the purpose of comparisons within the group of teenagers with PCHL on non-verbal ability and language, we used norms obtained from the HCG [15]. Each participant was assessed by a trained researcher, unaware of their audiological history. The group mean score and standard deviation score for a particular measure in teenagers in the HCG were used to derive age-adjusted z scores for the teenagers with PCHL on that measure.

#### 2.3.1 Receptive language

The Test for Reception of Grammar Version 2 (TROG) [24] was used to assess participants' receptive skills for spoken English grammar, and the British Picture Vocabulary Scale Third Edition (BPVS) [25] provided a measure of their receptive skills for spoken English vocabulary. Both of these assessments had also been used to measure the participants' receptive language skills at primary school age. An aggregate measure of receptive language was obtained by averaging the z scores for the TROG and the BPVS.

#### 2.3.2 Expressive language

The Expression, Reception and Recall of Narrative Instrument (ERNNI) [26] provided a measure of participants' expressive spoken language skills. Participants were required to produce a narrative based on a series of picture cues, and then subsequently to reproduce that narrative without the support of the pictures. Their narrative outputs were scored according to the ERRNI manual to produce three scores: an Initial score for the quality of their initial narrative, a Recall score for the quality of their recalled narrative, and a Mean Length of Utterance (MLU) score which reflected the average length of their utterances across both the initial and recall narratives. An aggregate measure of expressive language was obtained by averaging the z scores derived from the initial storytelling and recall scores from the ERRNI.

#### 2.3.3 Non-verbal ability

At Time 1 we assessed Non-Verbal Ability using the Raven's Standard Progressive Matrices [27]. At Time 2 the 20 min timed version [28] was used. Participants were given twenty minutes to work their way through a series of progressively more complex matrix reasoning puzzles. Raw scores reflecting the total number of correct items out of a possible 60 were calculated.

### 2.4 Statistical analysis

The primary outcome measure was specified as the Total Difficulties scores on the SDQ and group differences were tested with effect sizes obtained as standardised mean differences (SMDs) and associated 95% confidence intervals (95%CI). The distributions of the SDQ scores were somewhat skewed, as is usually found with this questionnaire [29]. To address this issue bootstrapped estimates of the standard errors were obtained [30]. A post-hoc power analysis indicated that, in a two-group comparison using a two-sided *t*-test, these sample sizes had 80% power to detect an SMD of 0.56 with alpha at 5%. The relationships between continuous measures, such as language scores, and the Total Difficulties score on the SDQ were tested using correlations. A post-hoc power analysis indicated that, using a two-sided test, the sample size of n = 72 had 80% power to detect a correlation of 0.32 with alpha at 5%.

## 3 Results

### 3.1 Characteristics of the samples

Of the 76 participants with PCHL and the 38 in the HCG in the current study, 73 (96%) and 37 (97%) provided parent-rated, 55 (72%) and 28 (74%) teacher-rated and 65 (85%) and 38 (100%) self-rated SDQ data respectively.

The teenagers with PCHL and the HCG were drawn from the same birth cohort and had similar baseline characteristics but those with PCHL were on average 0.74 years older than those in the HCG at the time of the teenage assessment (Table 1). However there were no significant correlations between age and scores on parent, teacher or self-rated SDQ Total Difficulties score in either the PCHL or HCG group.

**Table 1:** Characteristics of children in the PCHL group and the HCG.

	PCHL^a^ N = 76 Mean (SD)	HCG^b^ N = 38 Mean (SD)	Standardised mean difference (SMD) or odds ratio (95%CI)
Age at assessment (years)	17.09 (1.45)	16.35 (1.24)	SMD = 0.57 (0.20–1.29)
Non-verbal ability	−0.28 (0.83)	0.00 (1.00)	SMD = −0.30 (−0.70 to 0.10)
	**n (%)**	**n (%)**	
Female gender	37 (48.7)	13 (34.2)	OR = 0.55 (0.24–1.23)
English main language at home	70 (92.1)	36 (94.7)	OR = 1.54 (0.30–8.03)
Degree of hearing loss			
Moderate	33 (43.4)	–	n/a^c^
Severe/Profound	43 (56.6)	–	n/a
Other disability			
Cerebral palsy	2 (2.6)	0	n/a
Visual disability	2 (2.6)	0	n/a
Learning disability	13 (17.1)	1	OR = 7.69 (0.96–62.50)
None	62 (81.6)	37 (97)	OR = 8.35 (1.06–66.16)
Mother's education			
Less than A level	43 (56.6)	19 (50)	OR = 1.30 (0.60–2.85)
First cochlear implant			
Under age 8 years	11 (14.4)	–	n/a
After 8 years of age	3 (3.9)	–	n/a
Born in periods with UNHS^d^	37 (48.7)	–	n/a
PCHL confirmed ≤ 9 months	35 (46.1)	–	n/a

a PCHL Permanent childhood hearing loss.

b HCG Hearing comparison group.

c Not applicable.

d Universal neonatal hearing screening for PCHL.

There were no significant differences between the PCHL and HCG in terms of gender, English as the main language spoken by the family at home as reported by the parents, non-verbal ability or mothers education (see Table 1).

As expected, a higher percentage of children with PCHL (18.4%) than those in the HCG (3%) had one or more of cerebral palsy, visual impairment or learning disability (OR = 8.35, 95%CI 1.06 to 66.16) (Table 1). The SMD for Total Difficulties as rated by parents for teenagers with Other disabilities compared to those with none was +1.68, 95%CI 1.04 to 2.32. For this reason the results are reported below both for the entire PCHL group and separately for those without other disabilities.

### 3.2 Effects of attrition

To check on the possible biasing effect of selective attrition, those retained at follow-up, defined as a having a Time 2 score on parent-rated SDQ, were compared to those lost to follow-up on a range of measures at Time 1. For the PCHL group there were no significant differences between those retained and those lost to follow-up in terms of gender, mother's educational qualifications or English as the main language at home. There was no significant difference between these groups on the severity of hearing loss at Time 1. There were no significant differences in expressive and receptive language scores and nonverbal ability in those in the PCHL group at Time 1 and those lost to follow-up. Lastly there were no significant differences in the group mean parent-rated Total Difficulties scores at Time 1 between those in the PCHL group who were followed up and those lost to follow-up (SMD = −0.17, 95%CI −0.55 to 0.20).

For the HCG those lost to follow-up did not differ significantly from those retained on gender, mother's educational qualifications and English as the main language at home. The lost to follow-up and retained groups had similar parent-rated Total Difficulties scores at Time 1 (SMD = −0.12, 95%CI −0.63 to 0.38) and therefore on the variable of central concern in the analyses in the present paper there was no significant difference.

### 3.3 Comparison of the PCHL group and HCG on total difficulties scores in adolescence

On parent-rated SDQ, the PCHL group had significantly greater Total Difficulties scores than the HCG (SMD = +0.39, 95%CI 0.00 to 0.79) (F = 4.23, df = 1,106, p = 0.04) (Table 2). There were no main effects of gender (F = 0.03, df = 1,106, p = 0.87). The interaction between gender and group was not significant (F = 0.98, df = 1,106, p = 0.32). For teacher rated SDQ there was no significant difference between the PCHL group and the HCG (SMD = +0.17, 95%CI −0.28 to 0.62) (F = 1.04, df = 1,80, p = 0.31). On teacher ratings males scored more highly than females (F = 5.89, df = 1,80, p = 0.02). The interaction between group and gender for teacher ratings was not significant (F = 0.11, df = 1,80, p = 0.74).

**Table 2:** Mean and SD of SDQ Total Difficulties scores for adolescents with PCHL and the HCG by gender.

	PCHL^a^			HCG^b^				
	n	Mean	SD	n	Mean	SD	Standardised mean difference (SMD)	SMD 95%CI
**Parent-rated**								
Both sexes	73	8.48	6.17	37	6.22	4.95	+0.39	0.00 to 0.79
Females	35	9.00	6.21	13	5.31	4.27	+0.64	0.00 to 1.29
Males	38	8.00	6.18	24	6.71	5.30	+0.22	−0.29 to 0.73
**Teacher-rated**								
Both sexes	55	6.25	5.46	29	5.38	4.62	+0.17	−0.28 to 0.62
Females	24	4.83	5.30	10	3.20	3.94	+0.33	−0.41 to 1.07
Males	31	7.35	5.42	19	6.53	4.26	+0.16	−0.40 to 0.73
**Self-rated**								
Both sexes	65	9.74	5.18	38	9.13	5.14	+0.12	−0.28 to 0.52
Females	32	8.94	5.80	13	9.00	5.31	−0.01	−0.66 to 0.63
Males	33	10.52	4.44	25	9.20	5.16	+0.28	−0.24 to 0.79

a PCHL Permanent childhood hearing loss.

b HCG Hearing comparison group.

There was no difference in self-rated group mean Total Difficulties between the PCHL and the HCG groups (SMD = +0.12, 95%CI −0.28 to 0.52) (F = 0.33, df = 1,99, p = 0.56) (Table 2). There was no significant effect of gender (F = 0.36, df = 1,99, p = 0.42) nor a significant interaction between gender and group (F = 0.40, df = 1,99, p = 0.53) on self-reported SDQ Total Difficulties scores.

For those teenagers with PCHL and no disabilities the SMD compared to the HCG on parent-rated SDQ Total Difficulties was smaller and no longer significant (SMD = +0.21 95%CI −0.21 to 0.62).

### 3.4 Comparison of the PCHL group and national norms on total difficulties scores in adolescence

The mean scores for the HCG in Table 2 are somewhat lower than those in a large normative sample [31]. One sample t-tests were carried out to test whether the PCHL group had scores that were significantly different from these norm values (11–15 year olds - parent rated SDQ mean = 8.2, SD = 5.8; - teacher rated SDQ mean = 6.3, SD = 6.1). Neither the parent rated mean score (t = 0.39, df = 72, p = 0.70) nor the teacher rated mean score (t = 0.06, df = 54, p = 0.95) showed a significant difference from the mean of the norm group.

### 3.5 PCHL and HCG differences on specific types of EBD in adolescence

The differences in means between teenagers with PCHL and the HCG were not significant on the EBD sub-scales of the parent-rated SDQ (Multivariate F = 1.25, df = 4, 105, p = 0.30). This pattern of results remained unchanged when the analysis was limited to those without other disabilities.

There were no significant differences between the PCHL and HCG teenagers on the parent-rated SDQ Prosocial scale (SMD = +0.21, 95%CI −0.19 to 0.61). When the analysis was limited to those without other disabilities, those with PCHL had significantly higher Prosocial scores than the HCG (SMD = +0.64, 95%CI 0.22 to 1.06).

Teenagers with PCHL were not reported to have more difficulties than the HCG teenagers on the EBD sub-scales of the teacher-rated SDQ (Multivariate F = 0.25, df = 4, 79, p = 0.91). This pattern of results remained unchanged when the analysis was limited to those without other disabilities.

There were no significant differences between the PCHL and HCG teenagers on the teacher-rated SDQ Prosocial scale (SMD = −0.0.09, 95%CI −0.54 to 0.36). This analysis was unchanged when it was limited to those without other disabilities.

Teenagers with PCHL reported significantly higher overall scores than the HCG on the self-rated EBD sub-scales (Multivariate F = 3.32, df = 4, 98, p = 0.01). This arose from the high score on the Peer Problems sub-scale (SMD = +0.54, 95%CI 0.13 to 0.95) (F = 6.98, df = 1, 101, p = 0.01). The other sub-scales showed no significant differences. This pattern of results remained unchanged when the analysis was limited to those without other disabilities.

There were no significant differences between the PCHL and HCG teenagers on the self-rated SDQ Prosocial scale (SMD = +0.07, 95%CI −0.33 to 0.47). This result remained unchanged when the analysis was limited to those without other disabilities.

### 3.6 Changes with age in EBD as measured by parent-rating in the PCHL group and the HCG

The most appropriate way to examine longitudinal changes is to investigate groups for whom parent report is available in both childhood and adolescence for the same participants. Applying this to the 72 participants with PCHL for whom parent report was available at both time points, the mean Total Difficulties score showed no significant change from 9.22 to 8.29 (SMD = −0.16, 95%CI −0.48 to 0.17) (Table 3). The stability of individual differences in the Total Difficulties score is indicated by the correlation (r) between time points of 0.52 (95%CI 0.32 to 0.68).

**Table 3:** Mean and SD SDQ parent-rated Total Difficulties and sub-scale scores for adolescents with PCHL and the HCG at Time 1 (6–10 years) and Time 2 (13–20 years).

	PCHL^a^								HCG^b^							
		Time 1		Time 2						Time 1		Time 2				
	n	Mean	SD	Mean	SD	SMD^c^ 95%CI	Paired^d^*t*-test	r	n	Mean	SD	Mean	SD	SMD^c^ 95%CI	Paired^d^*t*-test	r
Total difficulties	72	9.22	5.81	8.29	6.00	−0.16 −0.48 to 0.17	t = 1.36,df = 71,p = 0.18	0.52	37	6.49	3.73	6.22	4.95	−0.06 −0.51 to 0.39	t = 0.38,df = 36,p = 0.71	0.54
Emotional Symptoms	72	1.67	1.70	1.92	1.95	+0.14 −0.19 to 0.46	t = 1.05,df = 71,p = 0.30	0.40	37	1.54	1.68	1.30	1.61	−0.15 −0.60 to 0.31	t = 0.81,df = 36,p = 0.42	0.39
Conduct Problems	72	1.60	1.62	1.21	1.36	−0.26 −0.59 to 0.07	t = 1.86,df = 71,p = 0.08	0.24	37	0.86	0.95	1.03	1.28	+0.15 −0.31 to 0.61	t = 0.76,df = 36,p = 0.45	0.35
Hyperactivity	72	4.43	3.12	3.24	2.59	−0.42 −0.74 to −0.08	t = 3.66,df = 71,p < 0.001	0.54	37	3.03	2.24	2.59	2.17	−0.20 −0.66 to 0.26	t = 1.15,df = 36,p = 0.26	0.47
Peer problems	72	1.53	1.76	1.93	1.77	+0.23 −0.10 to 0.55	t = 1.91,df = 71,p = 0.06	0.49	37	1.05	1.60	1.30	1.85	+0.14 −0.31 to 0.60	t = 0.81,df = 36,p = 0.42	0.45
Prosocial behaviour	72	7.58	2.38	8.43	2.19	+0.37 0.04 to 0.70	t = 3.42,df = 71,p = 0.001	0.58	37	8.38	1.42	7.89	1.47	−0.34 −0.80 to 0.12	t = 2.64,df = 36p = 0.01	0.70

a PCHL Permanent childhood hearing loss.

b HCG Hearing comparison group.

c Based on means and SD; this gives a smaller effect size and wider confidence intervals than those derived from the value of t in the paired-test (Dunlap et al., 1996).

d p values based on bootstrapping with 1000 iterations.

In the 37 participants from the HCG in whom parent report was available at both time points, there was no significant change over time in the mean Total Difficulties score from 6.49 to 6.22 (SMD = −0.06, 95%CI −0.51 to 0.39) with r = 0.54 (95%CI 0.27 to 0.94).

The only problem scale to show a significant decline was Hyperactivity for those with PCHL (SMD = −0.42, 95%CI −0.74 to −0.08). For the PCHL group there was a non-significant tendency (p < 0.06) to show an increase over time in Peer problems (SMD = +0.23, 95%CI −0.10 to 0.55). The Prosocial scale showed a significant increase in those with PCHL (SMD = +0.37, 95%CI 0.04 to 0.70) but no significant change in the HCG (SMD = −0.34, 95%CI −0.80 to 0.12).

The pattern of the results presented in Table 3 was unchanged when the analysis was limited to those without other disabilities.

The difference between the parent-rated Total Difficulties scores for the PCHL group and the HCG in this longitudinally studied sub-sample declined from SMD = +0.52 (95%CI 0.12 to 0.93) at Time 1 to SMD = +0.36 (95%CI −0.03 to 0.76) at Time 2. However this change in the difference is not significant in a repeated measures ANOVA (F = 0.37, df = 1,107, p = 0.54).

### 3.7 Cognitive and language abilities and EBD in the PCHL group and the HCG in adolescence

The PCHL group had poorer receptive language abilities than the HCG [13]. The correlation between receptive language and parent-rated Total Difficulties scores was significant for both the PCHL group (r = −0.32, 95%CI −0.53 to −0.02) and HCG (r = −0.33, 95%CI −0.59 to −0.01). For expressive language the correlations with parent-rated Total Difficulties scores were lower and not significant (PCHL r = −0.02, 95%CI −0.28 to 0.24, HCG r = 0.12, 95%CI −0.21 to 0.43). For non-verbal ability there was a significant correlation for the HCG only (PCHL r = −0.01, 95%CI −0.26 to 0.24; HCG r = −0.44, 95%CI −0.66 to −0.13).

Receptive language ability was therefore tested as a factor potentially accounting for the PCHL/HCG difference in Total Difficulties. An ANOVA showed a marginally non-significant effect of group (i.e. PCHL/HCG) on Total Difficulties score (F = 3.75, df = 1,108, p = 0.05). When receptive language was entered as a covariate the effect of group was no longer significant (F = 0.12, df = 1,95, p = 0.73).

Within the PCHL group those with severe/profound PCHL showed receptive language ability scores below those with moderate hearing impairment but this was not significant (p = 0.08) (SMD = +0.44, 95%CI −0.06 to 0.94). As a further check on the relationships between language, hearing loss and EBD, the correlation between Total Difficulties scores and receptive language ability scores was calculated within the moderate and severe/profound PCHL groups separately. These correlations were negative in each case; the lower the receptive language score, the higher the EBD score. The correlation with parent-rated Total Difficulties scores was significant for the moderate (r = −0.39, 95%CI −0.65 to −0.05) but not the severe/profound group (r = −0.21, 95%CI −0.53 to 0.17). Therefore the relationship between receptive language ability and EBD remains when severity of hearing impairment is taken into account, at least for the moderate hearing loss group. These results suggest that it is the difference in receptive language between the PCHL group and the HCG, rather than hearing impairment per se, that accounts for the effect of PCHL on EBD.

This analysis was repeated for those without other disabilities. Again the correlation with receptive language ability for parent-rated Total Difficulties was significant for the moderate hearing impairment group (r = −0.47, 95%CI −0.72 to −0.18) but not for the severe/profound impairment group (r = 0.13, 95%CI −0.26 to 0.49).

### 3.8 Factors within the PCHL group related to total difficulties scores

Parent-rated Total Difficulties scores were not significantly different between the participants with PCHL with and without a cochlear implant (SMD = +0.35, 95%CI −0.25 to 0.96). If the teenagers without a cochlear implant are compared on parent-rated Total Difficulties to other teenagers with severe/profound PCHL and a cochlear implant, the effect was more marked with those having cochlear implants having lower scores but was not significant (p = 0.08) (SMD = +0.61, 95%CI −0.08 to 1.29). There were 7% of those with other disabilities who received cochlear implants compared to 21% of those without other disabilities. This difference was not significant (OR = 3.45, 95%CI 0.41 to 28.85). This analysis was repeated for those without other disabilities and the pattern of results was unchanged. The difference between those with and without cochlear implants was not significant on parent-rated Total Difficulties scores (SMD = +0.14, 95%CI −0.50 to 0.77). The comparison with of those with a cochlear implant with other teenagers with severe/profound PCHL was also not significant (SMD = +0.35, 95%CI −0.38 to 1.07).

The SMD for parent-rated Total Difficulties in teenagers with severe/profound hearing loss compared to those with moderate hearing loss did not differ significantly for either the whole sample (SMD = +0.19, 95%CI −0.26 to 0.66) or those without other disabilities (SMD = +0.20, 95%CI −0.30 to 0.72).

Both receptive language ability and other disabilities were related to Total Difficulties scores. They are also closely related to each other. To test whether they are independently related to Total Difficulties scores, the correlations between receptive language ability and Total Difficulties score were calculated for the whole PCHL group (r = −0.32, 95%CI −0.53 to −0.07) and then for the group without other disabilities (r = −0.23, 95%CI −0.47 to 0.04). The latter correlation was corrected for restriction of range in the receptive language scores arising from this selection. The strength of relationship between receptive language ability and Total Behaviour score was reduced for those without other disabilities. This suggests that in part, the association between other disabilities and a high EBD score is mediated via poorer receptive language development.

### 3.9 Factors within the PCHL group without other disabilities related to Prosocial behaviour scores

Those without other disabilities in the PCHL group had higher Prosocial behaviour scores on parent ratings than the HCG and the mean Prosocial score for this group increased significantly from Time 1 to Time 2. As with Total Difficulties score, a number of factors were therefore examined to determine if they related to variation in Prosocial behaviour scores in this sub-group of the participants with PCHL.

At Time 1 (SMD = 0.62, 95%CI 0.09 to 1.13) but not at Time 2 (SMD = 0.34, 95%CI −0.16 to 0.86) there were significantly higher parent rated Prosocial behaviour scores in females compared to males. A repeated measures ANOVA showed there to be significant effect of age (F = 9.55, df = 1,57, p = 0.003) and gender (F = 5.44, df = 1, 57, p = 0.023) but no significant interaction between age and gender (F = 1.24, df = 1,57, p = 0.269).

At Time 1 (SMD = −0.13, 95%CI, −0.75 to 0.48) and at Time 2 (SMD = −0.37, 95%CI −1.00 to 0.27) there were no significant differences in the Prosocial behaviour scores rated by parents for those with and without cochlear implants. This pattern of results for cochlear implantation was unchanged if the analysis was restricted to those with other disabilities and severe/profound degrees of hearing loss.

The mean parent rated Prosocial score at Time 1 for those with moderate was significantly greater than that for those with severe/profound hearing loss (SMD = 0.58, 95%CI 0.07 to 1.91) at Time 1 but not at Time 2 (SMD = 0.48, 95%CI −0.05 to 1.00). A repeated measures ANOVA showed there to be significant effect of age (F = 9.50, df = 1,57, p = 0.004) and degree of hearing loss (F = 5.98 df = 1, 57, p = 0.018) but no significant interaction between age and degree of hearing loss (F = 0.28, df = 1,57, p = 0.600).

There was no significant correlation between receptive language score and parent rated Prosocial scores at Time 1 (r = 0.07, 95%CI −0.20 to 0.33) or Time 2 (r = −0.04, 95%CI −0.30 to 0.23).

These analyses suggest the only factors related to high Prosocial behaviour scores in the PCHL without other disabilities group were age, female gender and less severe hearing loss.

### 3.10 UNHS, early confirmation of hearing impairment and the risk of EBD

The cohort study from which these participants were drawn was originally designed to examine whether the provision of UNHS for hearing impairment improved the outcome for children with PCHL. Some of the children were born in periods when UNHS was available and others not. We found that in adolescence there was an effect of exposure to UNHS on EBD by parent-ratings (SMD = +0.48, 95%CI 0.01 to 0.95): the scores of those born during periods of UNHS were lower than the scores of those born in periods with no UNHS (Table 4). When severity of hearing loss was added as a factor the effect of UNHS remained significant (F = 4.26, df = 1, 73, p = 0.04). The effect of exposure to UNHS on parent-rated SDQ Total Difficulties was unaffected when the comparison was limited to those without other disabilities (SMD = +0.52, 95%CI 0.00 to 1.04). Those born in periods with UNHS tended to have higher receptive language scores (SMD = −0.25 95%CI −0.75 to 0.23) but this was not significant. For the PCHL group as a whole, receptive language scores were related to parent-rated SDQ Total Difficulties scores. When receptive language ability was added as a covariate the effect of UNHS was no longer significant (SMD = +0.42, 95%CI −0.08 to 0.93). The possible beneficial of exposure to UNHS on behaviour was therefore at least partially explained by the higher receptive language scores in this group.

**Table 4:** Mean parent-rated SDQ Total Difficulties scores by exposure to Universal Newborn Hearing Screening and early/late confirmation of Permanent Childhood Hearing Loss (PCHL) in total PCHL sample and in those without other disabilities.

	No UNHS			UNHS							
	N	Mean	SD	N	Mean	SD	SMD	95%CI	t	df	p
Total PCHL sample	38	9.87	5.81	35	6.97	6.28	+0.48	0.01 to 0.95	2.05	71	0.04
No other disabilities	29	8.14	4.72	30	5.53	5.26	+0.52	0.00 to 1.04	2.00	57	0.05

	Early confirmed			Late confirmed							

N	Mean	SD	N	Mean	SD	SMD	95%CI	t	df	p

Total PCHL sample	34	9.50	7.21	39	7.59	5.03	+0.31	−0.15 to 0.77	1.33	71	0.19
No other disabilities	26	7.00	5.79	33	6.67	4.63	+0.06	−0.45 to 0.58	0.25	57	0.80

PCHL = permanent childhood hearing loss; SD = Standard Deviation; SMD = Standardised Mean Difference.

UNHS= Universal newborn hearing screening.

Early confirmation of PCHL (i.e. by age 9 months) compared to late confirmation did not have a significant effect on parent-rated Total Difficulties (SMD = +0.31, 95%CI −0.15 to 0.77) (Table 4). The percentage of early and late confirmed participants with severe/profound impairment was 47.4% and 49.2% respectively (OR = 1.19, 95%CI 0.45 to 1.90) and the percentage of early confirmed participants with other disabilities was 22.9% compared to 14.6% of late confirmed participants (OR = 1.72, 95%CI 0.54 to 5.57). The effect of early confirmation on parent rated SDQ Total Difficulties at Time 2 remained not significant when restricted to those without cochlear implants (SMD = 0.25, 95%CI −0.03 to 0.77). The effect of early confirmation remained non-significant when the analysis was restricted to those without other disabilities (SMD = +0.06 95%CI −0.45 to 0.58). It also remained not significant when severity of hearing loss was added as a factor (F = 1.91, df = 1,73, p = 0.171). The lack of an effect of early confirmation was therefore not attributable to a confound with severity of hearing impairment (i.e. more severe impairment leads to early confirmation).

### 3.11 Age related changes in the effects of age at confirmation and exposure to UHNS

There is a relationship between age at confirmation and UNHS with early confirmation being more common when UNHS is in place (66% vs. 34%, OR = 3.69, 95%CI 1.42 to 9.52). To examine their joint relationship with parent-rated Total Difficulties a 2 × 2 ANOVA was conducted with age at confirmation and UNHS as factors and Total Difficulties score as the dependent variable. This analysis was repeated for behaviour at Time 1 (childhood) and at Time 2 (adolescence) for those participants with parent-rated SDQ scores at both time points (N = 72). The analysis was repeated excluding participants with other disabilities as the presence of such disabilities may have led to early confirmation and therefore distort the findings on the effects of early confirmation of hearing impairment per se.

**Fig. 1:**
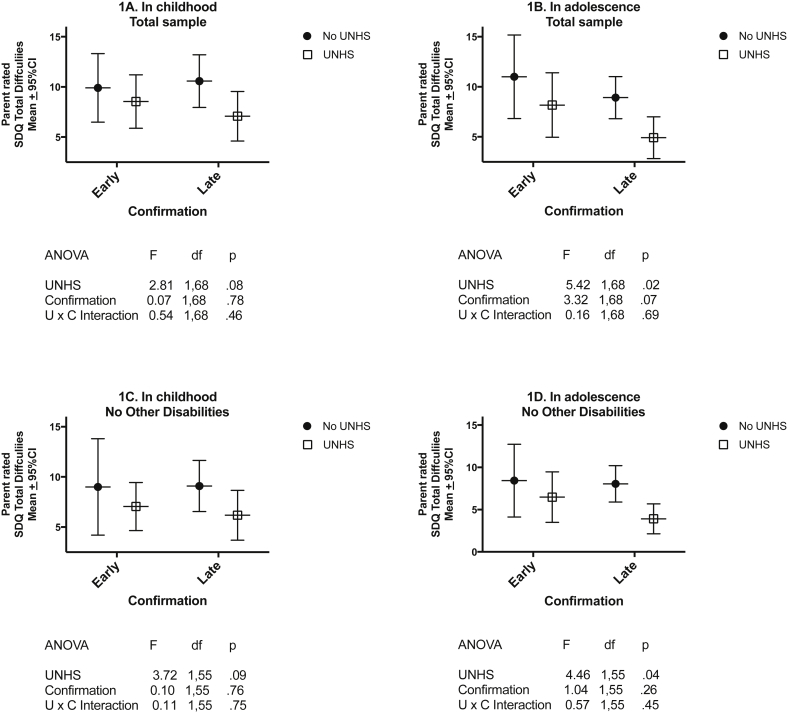
Mean parent-rated SDQ Total Difficulties score and 95%CI in childhood and adolescence for early/late confirmed and UNHS/No UNHS groups (UNHS = Universal newborn hearing screening).

These results are presented in Fig. 1. The pattern of means was similar in childhood (Fig. 1A) and in adolescence (Fig. 1B). The effect of UNHS was significant in adolescence for the Total sample (Fig. 1B) and for those with no other disabilities (Fig. 1D). The effect of age of confirmation was marginally non-significant in adolescence (p < 0.07) but not significant in childhood.

## 4 Discussion

Compared to teenagers with normal hearing, the teenagers with bilateral permanent childhood hearing loss in this study showed significantly greater emotional and behaviour difficulties scores in late adolescence, but only as reported by their parents. For teachers' and self reports these differences fell short of significance. It should be noted however that the study had less power to detect effects on teacher-rated behaviour as there were fewer participants for whom this measure was available. This suggests that the elevated rate of emotional and behavioural problems previously reported by parents in children with PCHL is also found in adolescence, although the mean Total Difficulties scores in both the PCHL group and the HCG were lower in adolescence compared to that in childhood in this longitudinal study; the difference between those with PCHL and the HCG group fell from 0.52 to 0.36 SDs between middle childhood and the late teenage years. Correlations indicated moderate stability in EBD scores between childhood and adolescence in those with PCHL.

The elevated scores relative to the HCG on the parent-rated EBD measure need to be put in the context of the number of teenagers with PCHL who show abnormally high SDQ Total Difficulties scores. On the parent-rated SDQ, borderline and abnormal scores are designated by a score of 12 or higher. In the PCHL group 21.9% had scores at this level. This suggests that the majority of teenagers with PCHL do not have EBD. Additionally, the presence of other disabilities substantially increased the Total Difficulties score. Once those with other disabilities were excluded the size of the difference in parent-rated EBD between the PCHL group and the HCG was greatly reduced and no longer significant. As well as factors directly associated with the disability, it should be noted that there are other factors, such as increased family stresses, possibly contributing to the vulnerability to EBD for those with other disabilities [32].

On the individual SDQ subscales, the PCHL group showed significantly higher self-rated Peer Problems than the HCG, though this difference was not significant for parent ratings on this subscale. It is also interesting to note that at Time 2 those with PCHL showed a higher level of parent-rated Prosocial behaviour than the HCG, but only for those without other disabilities. The presence of moderate rather than severe/profound degrees of hearing loss was also related to higher Prosocial behaviour scores. These results for Prosocial behaviour are at variance with the findings from a meta-analysis of SDQ sub-scales scores in those with a hearing loss [2]. In that analysis of the results of 10 studies using the parent rated SDQ there was a significant difference in the opposite direction with those with hearing loss having lower Prosocial scores than hearing children (SMD = 0.30, 95%CI 0.08 to 0.52) (N.B. the SMD reported in the meta-analysis had a reversed sign to that used here). In that meta-analysis, norms for the SDQ from the UK population [31] were used for comparison with the HL groups. If this comparison is used for the group without other disabilities in the present data set, the effect size (95%CI) falls from a significant SMD = 0.64, (0.22–0.1.06) to a non-significant SMD = 0.16 (−0.09 to 0.42). This disparity might also in part be due to the inclusion of children with other disabilities in the HL group in the meta-analysis.

A meta-analysis of SDQ reports on children with HL reported a similar pattern to that seen in the present study with higher Total Difficulties mean scores on parent and teacher reports, but not on self-reports, and elevated scores were reported for Peer Problems by parents, teachers and self-ratings [2]. The standardised mean difference obtained here can be compared with the above meta–analysis which reported SMDs of 0.23 and 0.34 for the differences between children with PCHL and the HCG on parent- and teacher-rated SDQ in 12 studies of children with hearing loss and hearing children. The equivalent SMDs in the present study were 0.39 and 0.17.

A feature of the results of this study is the association of the presence of other disabilities with an increase in the EBD scores in those with PCHL. A study of 140 adolescents with cochlear implants also reported a similarly large effect of additional disabilities on EBD [33]. In that study the adolescents with CI had significantly higher scores than a normal hearing comparison group on the peer problems SDQ sub-scale as rated by parents, teachers and on self-ratings. The risk of EBD was highest if the adolescent also had “risks” additional to cochlear implantation. These risks included general learning disorders, visual impairment and inner ear malformations. On parent and teacher ratings those with additional risks had significantly higher scores than the normal hearing group on both hyperactivity and conduct problems. Peer problems were reported more frequently on self-ratings by adolescents with CI than by those with normal hearing both with and without additional risks.

In the present study, as when the participants were examined in childhood [10], the presence of poor receptive language ability was a key risk for a high EBD score in those with PCHL. Indeed when an adjustment was made for the effect of receptive language ability, the PCHL group no longer had significantly higher SDQ scores compared to HCG. We conclude that the effects of poor receptive language ability accounted for the PCHL and HCG differences in EBD rather that hearing loss per se. The results also suggest that poor receptive language ability account at least in part for the high EBD score in those with other disabilities.

The finding of a benefit of UNHS on behaviour in adolescence contrasts with the absence of benefit reported in a recent study by Wake et al. [34]. That study investigated a range of cognitive and behavioural outcomes at ages 5–7 years in three Australian populations with contrasting approaches to the detection of bilateral congenital hearing loss. They used the same measure of EBD as that adopted here and the normative value of parent-rated Total Difficulties used in their analysis (6.9) was close to that found for the HCG (6.2). However, the mean Total Difficulties score for those with UNHS was higher (9.6) than that obtained for those with UNHS in the present study (7.0). One feature that differentiates the two studies is age at follow-up. The Australian sample was assessed at 5–7 years of age, while in the present study the participants were 13–20 years old. To test whether this age difference might account for the difference found for the effect of UNHS on behaviour the EBD scores for the children in the present study at age 5 0.5–11.5 years were examined. At that age, the effect of UNHS on SDQ parent-rated total difficulties scores was SMD = 0.34 (95%CI −0.12 to 0.80) and not significant. This is lower than the significant value of SMD = 0.48 (95%CI 0.01 to 0.95) obtained during the teenage years. This raises the possibility that the effect of UNHS on behaviour may become more marked with age.

However, the finding that the effect of UNHS on EBD in adolescence appears greater than that of early confirmation of PCHL is unexpected. We would predict that any effects of UNHS exposure on behaviour would be mediated via the benefits to language that result from early confirmation. An alternative explanation suggested by the absence of a significant benefit of early confirmation on behaviour in this sample may be that the benefit associated with UNHS that we observed was a sample-specific chance effect. Examination of the effects of UNHS and early confirmation on EBD outcomes when the Wake et al. cohort reaches adolescence would provide additional valuable insights into this issue.

The study reported here had a number of limitations. First, the annual attrition rate of 4% per annum over the 9 years since assessment at primary school among children with PCHL eligible for the present study limited its power to detect potentially clinical important effects. This attrition rate is nevertheless low for a longitudinal study of a teenage population with a long-term medical condition [35]. Moreover the EBD scores at age 8 years in those whose parents and teachers provided ratings in the present study were similar to those seen at 8 years in those that provided ratings only on in the assessment at primary school, indicating that there was not selective attrition related to the outcome measures of interest.

Second, given the finding that the presence of other disabilities was strongly related to EBD and that PCHL teenagers in this group were less likely to be in mainstream schools (19% vs. 77%), it was not possible to test for the suggested relationship between type of school attended and EBD [1] as this relationship would have been confounded by the presence of other disabilities.

Third, the teenagers with PCHL were slightly older than the HCG at assessment. However age showed no significant relationship with SDQ scores.

Fourth, a difficulty in interpreting the results stems from the low SDQ scores on parent and teacher ratings for the HCG compared to national norms [30]. Teenagers with PCHL had parent-rated SDQ scores that were significantly higher than the HCG. However their mean score was not significantly higher than the national norms on this measure. The comparison of the PCHL group with the HCG has the advantage that the groups are matched for the date and place of birth and the context in which the SDQ scores were obtained, but from these two comparisons there is ambiguity concerning the extent to which adolescents with PCHL have elevated parent-rated SDQ scores.

The study also had some strengths. The sample of children with PCHL was drawn from a geographically defined population base. The participants that were born in periods with UNHS were closely comparable with those born in periods without UNHS with respect to place and date of birth and audiological service provision other than UNHS. The participants were studied longitudinally from birth up to their late adolescent years. The HCG was identified from children born in the same hospitals as the PCHL and of similar age at assessment. Interviewers who undertook the assessments were blind to the UNHS status of the participants.

## 5 Conclusions

The present study is consistent with the conclusion that children and adolescents with PCHL are at increased risk of emotional and behavioural difficulties, as previously suggested by a meta-analysis. It also extends that conclusion by identifying the factors predisposing children with PCHL to EBD and by showing their developmental trajectory over time. More specifically, it suggests that in addition to PCHL, the presence of other disabilities and poor receptive language abilities create this vulnerability to EBD. In addition, those with EBD previously identified nine years earlier, are particularly likely to show EBD in adolescence. The continuity of EBD over time in the PCHL sample indicates that the long-term mental health of this group of children may particularly benefit from interventions in middle childhood and that such interventions should focus on those with poor receptive language. However, it is important also to recognise that, like hearing adolescents, the majority of adolescents with PCHL will not show clinically significant emotional and behaviour difficulties.

## Trial registration

ISRCTN03307358.
